# PERCEPTIONS OF LONG-TERM CARE RESIDENTS AND THEIR FAMILY MEMBERS ABOUT USING THE CONVERSATION STARTER KIT

**DOI:** 10.1093/geroni/igz038.562

**Published:** 2019-11-08

**Authors:** Sharon Kaasalainen, Tamara Sussman

**Affiliations:** 1 McMaster University, Burlington, Ontario, Canada; 2 McGill University, Montreal, Quebec, Canada

## Abstract

The need for a palliative approach in long term care (LTC) is widely recognized. However, advance care planning (ACP) is still rare. The purpose of this study was to explore the perceptions of LTC residents and their families about using an ACP tool called The Conversation Starter Kit (CSK). This study utilized a mixed methods approach. Data was collected in four LTC homes in Ontario, Canada from 31 residents and family members during an interview after they had completed the CSK. Data was analyzed using thematic analysis and descriptive statistics. All participants read all sections but only 73% completed all sections of the toolkit. Participants spent an average of 36 minutes discussing it with their family members and/or LTC staff. Participants reported: a better understanding of ACP after using the tool (80%), that the tool helped clarify the available resources and/or choices (53%), and that they felt less apprehensive about ACP after using the tool (60%). Qualitative findings revealed many strengths (e.g., usefulness, ability to start difficult conversations, content and clarification), and weaknesses of the tool (e.g., redundant information, difficulty understanding the content and lack of information regarding medically assisted dying). Family members noted that the toolkit would have been helpful to receive earlier on in their family members’ disease trajectory, perhaps before being admitted into LTC. These study findings support the CSK for residents and family members to have ACP discussions in LTC. Future work is needed to evaluate the effectiveness of the tool with a larger sample.

